# 

**Published:** 1921-12

**Authors:** 


**Official Publications Received.**

Annual Report of the Medical Officer of Health of Bournemouth.

Seventh Annual Report of the General Board of Control for Scotland. Cmd. 1396.

Public Health Reports of the United States Public Health Service. Vol. 36, No. 37.

Report of the Health of the City of Liverpool during 1920. By the Medical Officer of Health.

Annual Report of the Chief Medical Officer of the Board of Education, 1920. (Cmd. 1522.) H.M. Stationery Office. 6s. net.

Annual Report on the Health of the City of Coventry. By E. H. Snell, M.D., Medical Officer of Health and School Medical Officer.

Washington Government Printing Office: "Annual Report of the Surgeon-General of the Public Health Service of the United States, 1920."

Annual Report upon the Health of Blackburn for 1920. By W. Allen Daley, M.D., Medical Officer of Health and School Medical Officer.

Annual Report for the Year 1920 on the Sanitary Condition and Vital Statistics of Poplar. By F. W. Alexander, Medical Officer of Health.

St. George's Hospital must be congratulated on the excellent result of the Sale of Work and Pound Days organised by its nursing staff towards the end of October. The staff had aimed at obtaining the sum of £1,000, but the amount collected was no less than £1,998 net, while the value of the gifts in kind was over £32 more.

The Treasure Cot has earned an excellent reputation among mothers and nurses. Cosy, strong, and almost as compact and portable as a camp-stool, this folding cot is a most valuable possession where nursery space is restricted, when travelling with a baby, or when an extra cot is wanted for garden or balcony use. When draped it is charming to look at, and it is so constructed that the hammock portion can be easily detached for washing. Its makers, The Treasure Cot Co., Ltd., whose showrooms are at 124 Victoria Street, S.W. 1, have several other specialities, including the Treasure Crib, the Treasure Chair and Playtable, and three types of baby-pen or playground.

### OUR PRIZE LEAFLET.

In spite of our warning, some of our competitors gave us a good dose of cleanliness being next to godliness; but, strangely enough, no one took the trouble to explain the meaning of the adage, which is that even if you can't be good-looking you can at least be clean. One plagiarist, who is not apparently an early riser, treated us to the quotation that " I had my bath last night, so won't wash very much now "; and among the leaflets were several on the subject of uncleanliness. Someone, who, we think, is still happily at school, believes that " it is best to wash one's neck in the morning and clean one's ears too." We should have given him the prize if he had kept at that level. One competitor writes that this was a stupid subject to set for a leaflet, because there is so much that can be said about it which cannot be crowded into three hundred words. Several other of our leaflet writers found the same difficulty and exceeded the word-limit. Although some of these longer essays were admirable, we were bound to set them aside in fairness to those who took the trouble to fulfil our conditions. Some of those who were unsuccessful but received a " mention " last month sent in their efforts, and we hope they will continue until they are more fortunate. Among them *Fluffy* especially sent us a very pleasant leaflet, ruined, however, by the phrase, " the detergent action of soap," which few children would understand. We commend especially this month *Robin* (who should avoid lengthy Scriptural quotations with chapter and verse attached); *Calorie*, who sent us 302 words and a split infinitive; and a writer who sent neither name nor pseudonym, but had a pathetic reference to a pair (or brace) of guinea-pigs.

The following also did excellent leaflets: *Captain Kettle* (who, we hope, will enter for future competitions), *Anne Oviss*, *Health*, and *Christmas*.

We divide our prize of three guineas equally among *Spero*, *Pocrat*, and *Nota Bene*.

---

( 1 )

**Cleanliness.**

If you want to reach the State of Good Health you must, whatever else you may do, follow the Road to Cleanliness and take heed of the sign-posts on the way.

Sign-posts on the Road to Cleanliness.

No. 1.—Keep your Skin Clean.—Your skin is covered with millions of tiny pores through which poisonous waste matter from the body escapes. The pores soon get choked with dirt, and dirt leads to disease. To avoid this danger your whole body should be well washed with soap and water at least twice a week.

No. 2.—Keep your Hands and Nails Clean. Dirt and germs soon collect on the hands and under the nails. It is most important that you should never sit down to a meal without having first washed your hands and cleaned your nails.

No. 3.—Keep your Nose Clean by learning how to use a handkerchief correctly. You should always breathe through your nose, as by doing so the air becomes purified and warmed before reaching the lungs. Your nose cannot do its work properly if you do not keep it clean and free.

No. 4.—Keep your Teeth Clean by brushing them every morning and every night. If you do not do this poisonous matter will form in your mouth. Some of it will ruin your teeth, and some of it will find its way into your body.

No. 5.—Keep your Clothes Clean. Body lice love to live in dirty clothes. You should change your underclothes at least as often as you wash your body.

No. 6.—Keep your Food Clean by preventing dirt and flies from coming into contact with it.

No. 7.—Keep your Room Clean and leave your windows open day and night. A dirty, stuffy room is the home of many germs which cause disease.

*Nota Bene.*

( 2 )

**Cleanliness.**

Your Body is your House; you must live in it
ALL YOUR LIFE.
You are both Master and Servant in this house. So you have both to give orders and to carry them out. If you want your house to be
CLEAN,
COMFORTABLE, and
COMELY,
You must use plenty of
SOAP and WATER.
Never come down to breakfast without washing your hands and face, neck and ears.
Never eat your dinner, tea or supper with dirty hands.
Never go to bed with dirty teeth.
It is only the lazy people who are dirty.
IF YOU DO NOT LIKE A DIRTY HOUSE YOU CANNOT LIKE A DIRTY BODY.
You want your house to be a *beautiful* house.
It cannot be *beautiful* unless it is clean.
You want your house to be a *comfortable* house.
It cannot be *comfortable* unless it is clean.
There are THREE THIEVES which are always trying to get into that house.
They are
DIRT,
DISEASE, and
DEFORMITY,
and if once you let them in they will steal away those precious treasures of yours, which are
HEALTH and HAPPINESS.
You must keep them out AT ALL COSTS, for if you lose these two treasures you have nothing left with which to defend
YOUR LIFE,
and then the biggest thief of all will come and take that, and his name is Death.

*Spero.*

**The Prize Leaflets—(*cont.*).**

**( 3 )**

**Cleanliness.**

If dirt is matter in the wrong place, and disease is matter in the wrong condition, we must clearly keep clean if we would keep well.

Health is only a good habit—partly inherited and partly acquired.

To form, or maintain, the habit of health, your body needs: A clean outside, a clean inside, and a clean mind in control of them.

To keep clean outside nothing is better than the regular use of soap and water. Every day the whole body should be scrubbed. A hot bath on Saturday night is not enough. A cold bath every morning is also desirable.

A copper or wash-tub makes an excellent bath, and nowadays cold water is always available.

People who wash their faces, but not their feet or bodies, are as dirty as those who clean only the outside of their milk-jugs.

For a clean inside three things are needed: regular meals, regular exercise, and regular motions of the bowels.

Most people now have enough to eat, and regular exercise comes with regular work. These two will generally keep the bowels open; and what mother does not know the medicines occasionally needed?

A clean Outside and Inside will generally ensure a clean Mind also.

A clean Mind is given by plenty of Recreation and plenty of Work. Recreation means change of occupation. The more recreations you have, the merrier you will be.

Health means a Merry Mind in a Clean Body.

*Pocrat.*

### A Leaflet Competition

***We offer Three Guineas for the best new and original leaflet of not more than 300 words on***

**"EATING and HEALTH"**

The Leaflet must be suitable for circulation in the Public Elementary Schools and among the Scholars of the Secondary Schools. The result of this Competition will be announced and the successful Leaflet will be printed in the January Number of "The Hospital and Health Review." We reserve the right to republish any of the Leaflets submitted to us.

RULES AND REGULATIONS.

1. This Competition is open to all.
2. Leaflets must be written or typed on unprinted paper on one side of the page only.
3. Competitors must employ a pseudonym.
4. Leaflets must be received by the Editor at "The Hospital and Health Review," 28 Southampton Street, Strand, W.C. 2, by **the first post on December 12, 1921.** Envelopes must be marked in the top left-hand corner with the word "Leaflet."
5. The Editor's decision is final.

### RECENT OFFICIAL PUBLICATIONS.

[To be obtained at H.M. Stationery Office, Imperial House, Kingsway, W.C. 2. The prices include postage.]

STATUTORY RULES AND ORDERS, 1921.

No. 1,627. National Health Insurance Central Fund Regulations. October 26, 1921. 2d.

No. 1,633. Public Health, England. Notification of Infectious Diseases. The South Crosland (Measures) Regulations. November 1, 1921. 2d.

No. 1,657. Coal Mines. Precautions against Coal Dust. 2d.

No. 1,713. Factory and Workshop. Women and Young Persons (Employment in Lead Processes). November 8, 1921. 2d.

No. 1,714. Factory and Workshop. Women and Young Persons (Employment in Lead Processes). November 8, 1921. (Medical Examination.) 2d.

No. 1,715. Factory and Workshop. Women and Young Persons (Employment in Lead Processes). November 8, 1921. (Cloakroom Accommodation.) 2d.

Board of Education. Regulations for the Training of Teachers. Hygiene Syllabus. 4d.

Quarterly Return of Marriages, Births, and Deaths. England and Wales, No. 291. 2s. 7½d.

S.O.P. Instructions for the Physical Examinations of Recruits. 3d.

A leaflet describing the merits of the "Sterilendum" Enema has been issued by Messrs. J. G. Ingram & Son, Ltd., Hackney Wick, E. 9., who will be pleased to forward, post free, a copy to every applicant. The "Sterilendum" is wholly sterilisable, it being specially produced to meet the demand of the medical profession and others interested in hygiene.

